# What is the role of epistemic communities in shaping local environmental policy? Managing environmental change through planning and greenspace in Fukuoka City, Japan

**DOI:** 10.1016/j.geoforum.2019.04.024

**Published:** 2019-08

**Authors:** Leslie Mabon, Wan-Yu Shih, Kayoko Kondo, Hiroyuki Kanekiyo, Yuriko Hayabuchi

**Affiliations:** a School of Applied Social Studies, Robert Gordon University, Scotland, United Kingdom; b Department of Urban Planning and Disaster Management, Ming-Chuan University, Taiwan; c Faculty of Design, Kyushu University, Japan; d Global Innovation Center, Kyushu University, Japan

**Keywords:** Environmental governance, Environmental history, Epistemic communities, Fukuoka, Urban planning

## Abstract

This paper evaluates the role of epistemic communities in shaping local environmental policy, through the case of management of environmental change through planning and greenspace in Fukuoka City, Japan. Amidst increasing global interest in the role of evidence-based policy and urban science in responding to environmental issues in cities, Fukuoka is distinctive. Locally-situated scholars in Fukuoka have, for several decades, sought to shape local responses to environmental change by influencing policy for the built environment and greenspace. Through analysis of scholarly outputs produced by scholars working at universities and research institutes within Fukuoka and policy documentation produced by the city government, we characterise the development of Fukuoka’s urban environmental change epistemic community. We suggest that built environment and greenspace policy to respond to environmental change in Fukuoka has been shaped by an epistemic community in three ways. These are: (a) a common belief in techno-scientific evidence derived from empirical observation; (b) a shared interest in urban planning and greenspace as a vehicle for realising change; and (c) a common normative concern with citizen wellbeing, rooted in negative historical experiences with pollution. We argue that policy formation driven by scholarly expertise in cities may have a greater chance of taking root if there is a favourable historical context of locally-led environmental science research, personal investment of the epistemic community members in the city, and regular dialogue between the epistemic community and wider society in the city. We conclude that a strong and reflexive epistemic community, working in collaboration with environmental and civil society actors, is important in understanding an appropriate response to current urban environmental challenges.

## Highlights

• Assess role of techno-scientific expertise in governing urban environmental change.
• Evaluate evidence-driven built environment and greenspace policy in Fukuoka.
• Epistemic community shapes Fukuoka’s built environment and greenspace policy.
• Local history gives favourable context for environmental science in public interest.
• Findings nuance understanding of how epistemic communities work at urban scale.

## 1 Introduction

Climate change, sustainability and wider environmental issues are increasingly framed at the city level (Bulkeley, 2013, Parnell, 2016). Sustainable Development Goal 11 is devoted to sustainable cities and communities; UN Habitat’s New Urban Agenda puts cities at the heart of sustainable development; and a number of networks have emerged, including ICLEI – Local Governments for Sustainability, C40 Cities, and the Asian Cities Climate Change Resilience Network. Initiatives such as the Intergovernmental Panel on Climate Change’s Cities and Climate Change Science programme, and the Urban Climate Change Research Network, show academics are also gaining interest in the city as a site for contemporary environmental problems and solutions.

Within this turn, ‘evidence-based’ policy and governance is widely advocated to promote urban development compatible with climate change and sustainability imperatives. There is accompanying interest in the ‘science’ of cities; the people producing this science; and their relationship to city governance. For example, the New Urban Agenda (UN-Habitat, 2017); ICLEI’s reportage on its Resilient Cities conference series (ICLEI, 2018); and the output of an international expert panel on science and the future of cities endorsed by *Nature Sustainability* (Acuto et al., 2018) all refer to evidence-based governance and scientific knowledge for sound policy-making. Indeed, the idea of cities appointing chief science advisors or scientific advisory boards is raised in both the *Nature Sustainability* expert panel and a commentary piece coinciding with emergence of IPCC Cities (and authored by a number of members of the IPCC Cities Scientific Steering Committee) (Acuto, 2018, Baiet al., 2018).

City dwellers are at very real risk of harm from environmental changes such as increased instances of extreme heat, more intense rainfall and associated flooding, and declines in air quality. Guiding urban governance with expert knowledge of what constitutes technically and scientifically appropriate countermeasures is therefore highly desirable. Yet it is also crucial to reflect on the dynamics of how expertise from urban science communities influences environmental policy at the city level. This matters because extant scholarship on the role of expertise in governing the built environment suggests that in a particular locale, the flow of knowledge between science and practice is not objective or value-neutral, and can be informed by societal context. Acuto et al. (2018: 43) call for attention to the “political economy of expertise on cities.” This means considering questions such as whose knowledge shapes urban policies and how this expertise moves between cities and countries (Khirfan et al., 2013); who is defined as an ‘expert’ and how their claims to authority are made (Cashmore et al., 2015); and the possibility that expert knowledge might itself not be apolitical or closed (Ozdemir, 2018).

This paper therefore builds on emergent thinking on how epistemic communities – essentially, communities of techno-scientific experts working to inform policy through their knowledge – operate in the environmental sphere in cities as opposed to at the international level (Bahadur and Tanner, 2014, Hughes and Romero-Lankao, 2014). To do this, we take one city context – Fukuoka in Japan – and evaluate how and why evidence-based urban environmental policy can emerge for a specific issue. Fukuoka has several decades’ experience of producing scientific evidence locally, and using this knowledge to inform management of environmental change through built environment and greenspace planning. We assess how Fukuoka has come to host institutions with strong competence in researching the local environment, and how a community of experts from academia and research has shaped urban planning and greenspace policy in response to environmental change in the city. Within this, we pay particular – but not exclusive – attention to the relationship between the built environment, greenspace and urban thermal environments as an area in which Fukuoka has made early and notable progress with regard to evidence-based policy.

Fukuoka is distinct in that it engaged with local environmental change issues comparatively early in Japan. Local institutions have conducted applied research on the city’s climate since the mid-1980s. The city produced its first local climate change plan in 1994, earlier than other large cities in west Japan such as Kyoto in 1997; Kobe in 2000; and Osaka in 2002. Fukuoka’s city government has since then had sustained collaboration with academia and research institutes on issues of the built environment and environmental change. These have led to tangible changes in policy and practice (see Section 3). Through its subtropical climate and ongoing expansion, Fukuoka shares commonality with cities not only in Japan, but also in wider Asia. In practical terms, it is hence valuable to understand what has made evidence-based policy for the built environment, greenspace and environmental change relatively successful in Fukuoka. Based on the Fukuoka experience, we argue that local environmental policy formation driven by techno-scientific expertise may be more likely to produce tangible actions reducing citizens’ risk of harm under two conditions. First, if there is positive historical experience of expertise resolving local environmental issues. Second, if there is a local social, economic and political context which facilitates interaction between the epistemic community and wider society.

## 2 Epistemic communities and urban environmental policy

To assess how a community of experts has come to inform a specific area of environmental policy in Fukuoka over a period of several decades, this paper works with the concept of epistemic communities. An epistemic community is a group of scholars working together to shape policy through their knowledge. Lovell and MacKenzie (2011) explain the key concern of epistemic communities thinking is to consider how experts bring about change, especially in terms of the knowledge and expertise they possess. Haas (1992) argues an epistemic community can be defined by four characteristics: (a) shared normative belief, meaning the community members have similar kinds of values which guide their actions; (b) shared causal beliefs, which means the members generally agree how the natural world operates and what actions/policies are required to reach desired outcomes; (c) shared notions of validity, meaning community members have common criteria for assessing what counts as valid knowledge; and (d) a common policy enterprise, whereby members usually aspire to use their expertise to inform policy for the benefit of society. To assess the effectiveness of an epistemic community, one may wish to consider its ability to influence policy; its association with the wider public; and its success in communicating agendas more broadly (Stephens and Liu, 2012).

Dunlop (2017) holds that epistemic communities have most power in situations of high complexity and uncertainty, where stakeholder and decision-maker knowledge is limited. Epistemic communities scholarship initially focused on international networks (Toke, 1992). However, the significant body of literature around complexity and the involvement of multiple actors in the urban environmental change context (e.g. Bulkeley, 2013, Friend et al., 2014) indicates that the idea of epistemic communities may have some value in helping to evaluate where and why local environmental policy has been successful. Indeed, Bahadur and Tanner (2014) suggest that as cities are sites for concentration of intellectual capital, ‘localised’ epistemic communities reflecting the knowledges and policies appropriate to the local context may emerge. Harris and Moore (2013) too observe the role epistemic communities may play in urban policy transfer by helping to legitimise certain ‘best practices.’ At the city scale, Pieterse (2006) sees value of epistemic communities in being able to simultaneously understand and work within the rationales of mainstream government, yet also imagine alternatives. Yet Hughes and Romero-Lankao (2014: 1024) believe that when it comes to epistemic communities and urban climate change policy specifically, “relatively little work has focused on understanding science–policy interactions […] and what influences the choices cities make about science.”

The purpose of this paper is therefore to assess the value of the epistemic communities concept in understanding how environmental policy implementation driven by knowledge may emerge at the city scale. We do this by characterising the science-policy interface around environmental change management through the built environment and greenspace in Fukuoka City in Japan. We take up the challenge of Dunlop (2013) to consider how this epistemic community emerged and evolved over time. In doing so, however, we bear in mind Toke’s (1992) critical take on epistemic communities. That is, whilst epistemic communities are driven by knowledge based on empirical observation, when it comes to environmental issues, they may be just one interest group operating in a domain where perceptions over the ‘right’ course of action are inevitably driven by the social context (Toke, 1992). We also note Pieterse, 2006, Finewood, 2016 that epistemic communities may inadvertently converge towards policy status quo, and hence lose their ability to produce new or different forms of governance. There is also increasingly widespread understanding that a comprehensive response to urban environmental change needs to address questions of fairness and justice (e.g. Anguelovskiet al., 2016, Reckien et al., 2017). Accordingly, in Sections 5.3 and 6 we question the extent to which an epistemic community may be able to engage with some of these broader questions whilst retaining its scientific evidence base.

## 3 Background: Fukuoka City, environmental change and the built environment

### 3.1 What makes Fukuoka distinct? Geography, policy and expertise

**Fig. 1:**
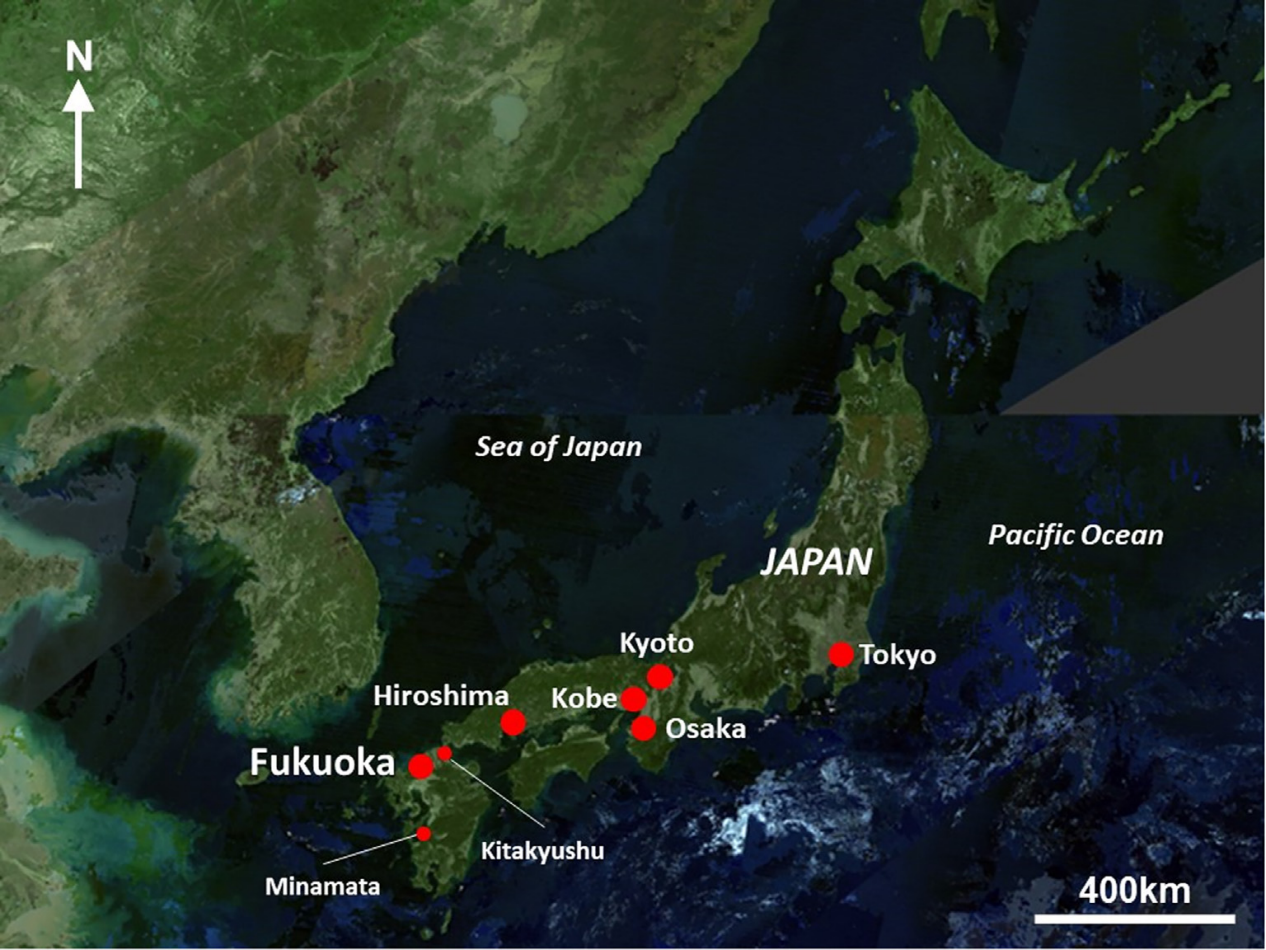
Location of Fukuoka City within Japan, showing major cities and locations mentioned in the text.

*Source:* adapted from Geospatial Information Authority of Japan, 2019.

**Fig. 2:**
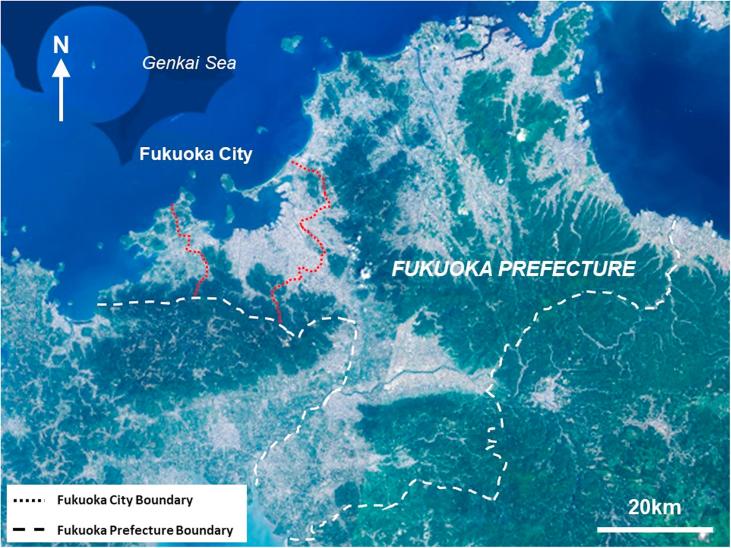
Topography of Fukuoka City and Fukuoka Prefecture.

*Source:* adapted from Geospatial Information Authority of Japan, 2018.

This section provides background to Fukuoka City, and identifies factors which make Fukuoka conceptually and practically valuable for understanding epistemic communities in local environmental policy. Fukuoka is the largest city on the southern island of Kyushu, Japan. Approximately 1.6 million people live within the Fukuoka City municipal area as of April 2019 (Fukuoka City Statistics, 2019). The larger Fukuoka Prefecture has a population of approximately 5 million people, around 2.5 million of whom are in the urbanised areas of Fukuoka and Kitakyushu (Fukuoka Prefecture, 2019). The city has a humid subtropical climate and is located on the Genkai Sea, which opens out into the Sea of Japan (see Fig. 1, Fig. 2). Fukuoka has the highest population growth rate (7.1% from 2010 to 2017) and the highest local tax revenue growth rate (4.2% between 2008 and 2015) of any major city in Japan (Fukuoka Asian Urban Research Center, 2018). There is significant regeneration in the central area of Fukuoka and expansion to the west.

Fukuoka’s actions on managing environmental change at the local level warrant particular attention. Fukuoka City produced its first local climate change plan – the Fukuoka City Local Climate Change Countermeasures Promotion Plan – in 1994. This made Fukuoka one of the first cities in Japan to do so, ahead of other early adopters such as Kyoto (1997) and Kobe (2000). Fukuoka’s consideration of climate change in urban planning and policy also came before key international organisations such as ICLEI and IPCC began to explicitly argue for local climate change action (see Section 5). This indicates the need for action on environmental change in Fukuoka was determined locally, rather than in response to wider global trends.

Fukuoka has a tradition of techno-scientific expertise from local institutions informing policy for managing environmental change (see Table 1, Table 2 in Section 5). Scientific research produced by local institutions has made recommendations for cooling and flood risk reduction in the city’s central area, and has informed Fukuoka’s greenspace plans since 1999 (Fukuoka City, 1999, Fukuoka City, 2009). Both the local and regional climate change plans in Fukuoka draw on input from academic expert committees. The resulting plans integrate mitigation and adaptation with the city’s broader environmental plan (Fukuoka City, 2016); and acknowledge the importance of ecosystem services in climate change responses (Fukuoka Prefecture, 2017). As well as participation in planning processes, local institutions in Fukuoka also have a long history of undertaking applied empirical research on environmental issues in the city. Of particular note is the work done in Fukuoka on the built environment, greenspace and the urban thermal environment. This includes a locally-written textbook into the role of greenspace in regulating the climate of a city (Nitta et al., 1981); studies into the urban thermal environment and wind patterns in the early 1990s with associated policy recommendations (Katayama et al., 1990, Katayamaet al., 1993); and the production of a Fukuoka urban climate atlas, a climate function map focusing on effects of green space on urban heat islands with an urban climate simulation system (Yoda and Katayama, 1998, Ichinose et al., 2003). Practice-focused research on flooding has also emerged more recently in the city (Takashi, 2013; Yamashita et al., 2016).

**Table 1:** Roles and actions of main institutions involved in urban environmental governance and policy in Fukuoka City.

Institution	Sector	Activity within community	Indicative studies/texts
Fukuoka Center for Climate Change Actions (FCCCA)	Civil society/regional government	Public engagement and knowledge dissemination; managed by KEEA and Fukuoka Prefecture	N/A
Fukuoka City Government – Environment Division	Local government	Knowledge synthesis - production of city climate change action plan	Fukuoka City Climate Change Action Plan (2016); 2003 study into urban heat
Fukuoka City Government – Housing and Planning Division	Local government	Knowledge synthesis - production of city urban plan; production of greenspace plans and technical guidance (via Green City Promotion Department); assessment of city green network	Fukuoka City, 1999, Fukuoka City, 2009, Fukuoka City, 2014
Fukuoka District Meteorological Observatory	Academia/research	Provision of regional climate data and predictions	Climate change predictions for Kyushu and Yamaguchi Prefecture based on IPCC A1B scenario (Fukuoka District Meteorological Observatory, 2014, Fukuoka District Meteorological Observatory, 2017)
Fukuoka Prefectural Government	Regional government	Knowledge synthesis - production of regional climate change action plan	Fukuoka Prefecture Climate Change Action Plan (2017)
Fukuoka University	Academia/research	Participation of academics in Fukuoka City and Fukuoka Prefecture Climate Change Expert Committees; membership of KEEA advisory board	1988 review into land use and the urban environment (Asano, 1988); 1992 paper on laws and finance for low-carbon local development (Asano, 1992a); 2017 text on UN Sustainable Development Goals (Asano, 2017); community and government engagement on urban flood resilience
Fukuoka Asian Urban Research Center	Independent research organisation	Knowledge synthesis – assessment of socio-economic data trends; evaluation of urban policy; dissemination of knowledge about Fukuoka to wider Asia	*Fukuoka Growth* report series for synthesis and analysis of socio-economic data; *Urban Policy Studies* periodicals
Kyushu Environmental Evaluation Association (KEEA)	Independent research organisation	Hosting of FCCCA; annual synthesis of research via local journals	*Environmental Evaluation* annual journals; 2006 study into urban heat
Kyushu Institute of Technology	Academia/research	Community and government engagement on urban flood resilience (e.g. Yamashita et al., 2016); collaboration with other institutions on urban thermal environment research	Yamashita et al. (2016) community and government engagement on urban flood resilience
Kyushu University/Kyushu Institute of Design (merged 2003)	Academia/research	Participation of academics in Fukuoka City and Fukuoka Prefecture Climate Change Expert Committees; production of academic texts	*Design of micro-weather in urban greening* book (Nitta et al., 1981); ongoing research into urban wind and thermal environments (e.g. Hagishima, 2018); community and government engagement on urban flood resilience

**Table 2:** Summary of content analysis: policies, research, messaging and proposed actions.

Time period	Relevant local policies in Fukuoka	Underpinning local research	Messaging and framing around urban environmental change	Proposed actions in research outputs to address environmental change through the built environment	Relevant international and national context for environmental change in cities
1980s	Fukuoka City Environmental Plan (1986)	Grant-in-Aid for Scientific Research: ‘Land Use and Sustaining and Improving the Environment in Cities and their Surroundings’ (1985–7); establishment of Fukuoka Urban Research Center (1988) (renamed Fukuoka Asian Urban Research Center in 2004)	Liveable environment / *kaiteki kankyou*	Creation of urban green master plan / greater role for urban planning (Sakagami, 1981, Asano, 1988)	*Our Common Future* (Brundtland Report) (1987)
1990s	Fukuoka City Local Climate Change Counter-measures Promotion Plan (1994); Fukuoka City Green Basic Plan (1999)	Early studies on climate analysis for urban planning in Fukuoka (Katayama et al., 1990); Grant-in-Aid for Scientific Research ‘For the reasonable arrangement of urban green spaces based on distribution and function of green spaces’ (1996–8)	Balancing environment and development towards urban sustainability	Maintenance of wind corridors and water bodies via planning; enhancing urban greening (Katayamaet al., 1991, Katayamaet al., 1993, Miura, 1995)	International Council for Local Environmental Initiatives (ICLEI) established (1990); UNFCCC adopted (1992); Agenda 21 and Rio Declaration (1992); 1994 Japanese-German meeting on climate analysis for urban planning, attended by Fukuoka-based researchers (Hoschele et al., 1995); UN Habitat II: Istanbul Declaration on Human Settlements and the Habitat Agenda (1996); Kyoto Protocol signed (1997)
2000s	Fukuoka City Local Climate Change Counter-measures Promotion Plan (2nd/3rd versions) (2001/2006); Fukuoka City New Green Basic Plan (2009)	Grant-in-Aid for Scientific Research ‘Fundamental study on improvement of green space contributing to reducing the influence of B ultraviolet rays in the outdoor living environment’ (2001–2); Fukuoka City Government study into urban heat (2003); KEEA/Kyushu University urban thermal environment observations (Tanaka, 2009, Tanaka et al., 2009)	Managing climate change at the local level within Fukuoka City	Development of evidence base via observation; prioritisation of areas for actions to improve comfort of lived environment (Tanaka, 2009, Yoda, 2009)	Establishment of C40 Cities Climate Leadership Group (2005); Japanese Government Act on Promotion of Global Warming Countermeasures (2008)
2010s	Fukuoka City Climate Change Action Plan (2016); Fukuoka Prefecture Climate Change Action Plan (2017) Fukuoka City Third Green Basic Plan (due 2020)	First *Fukuoka Growth* urban data report produced by Fukuoka Asian Urban Research Center (2013); Fukuoka District Meteorological Observatory regional predictions based on IPCC A1B scenario; university-government-citizen collaboration on flood resilience (Yamashita et al., 2016)	Local lived-in environment as fundamental basis for quality of life, climate adaptation, and sustainability; people living in harmony with nature	Development which respects and preserves natural environments (Ooi, 2008, Takashi, 2013); integrated climate change adaptation plans (Fukuoka City, 2016;Hijioka, 2017); consideration of ecosystem services within regional planning (Fukuoka Prefecture, 2017)	Launch of ICLEI Resilient Cities: World Congress on Cities and Adaptation to Climate Change (2010) UN SDG11: Sustainable Cities and Communities (2015); Paris Agreement (2015); UN Habitat III: New Urban Agenda (2016); IPCC Cities and Climate Change Conference (2018); Japanese Government Climate Change Adaptation Act (2018)

These actions are not unique to Fukuoka. Yet they are significant in practical terms for at least two reasons. First, despite extensive and well-established policies for disaster prevention (Hijioka et al., 2016), local governments in Japan have arguably been slow to develop policy for adaptation to contemporary environmental change, due to limitations in resourcing, lack of expert knowledge, and limited national government support (Kameyama, 2016, Babaet al., 2017). It is hence valuable to understand why Fukuoka, as a Japanese city, has been able to develop relevant comprehensive local policies at such an early stage. Second, Fukuoka’s experience with evidence-based policy and the built environment reflects emerging international research interests. IPCC Cities’ *Global Research and Action Agenda on Cities and Climate Change Science* (Prieur-Richard et al., 2018) argues that the vast majority of research into how urban micro-climates integrate into urban planning and design has come from North America, Europe and Australia. There is also growing interest in understanding how techno-scientific knowledge can be meaningfully integrated into urban- and greenspace planning for adaptation to environmental change (e.g. Meerow and Newell (2017) on Detroit; Mabon and Shih (2018) on Taipei). Given these broader trends, the activity of the epistemic community in Fukuoka around managing the city’s climate (especially urban thermal environments) via the built environment and greenspace are a focal point of this paper.

Conceptually, this early and sustained engagement of a community of environmental scholars with built environment and greenspace policy in Fukuoka can be used to empirically evaluate emerging thought on the role of epistemic communities at the city level (particularly with regard to environment and climate policies). Following Lane et al. (2008), Fukuoka provides an opportunity to assess the value of epistemic communities thinking when applied not in the transnational context, but in relation to networks operating within a specific country. Fukuoka also holds a number of characteristics typical of the kinds of cities where environmental change will be felt most, and towards which the rhetoric of knowledge-sharing and evidence-driven action outlined in Section 1 is directed. Fukuoka is (a) subtropical; (b) expanding in terms of population, area and economy; and (c) located outside of the North American, European and Australian context from which much knowledge to date on managing environmental change through the built environment has accrued (Prieur-Richard et al., 2018). Evaluation of the characteristics, effectiveness and limitations of the epistemic community in Fukuoka hence offers insight into how knowledge and scholarly expertise operates in urban environmental governance.

### 3.2 Insight into Fukuoka’s built environment science-policy interface: The Kyushu Environmental Evaluation Association and the *Environmental Evaluation* periodicals

To assess the development of Fukuoka’s urban environment epistemic community over time, we focus on the content of the annual *Environmental Evaluation* periodicals produced by the Kyushu Environmental Evaluation Association (KEEA). KEEA is an independent, legally-incorporated foundation headquartered in Fukuoka City. KEEA was founded in 1970 as the Kyushu Water Quality Analysis Research Institute, then renamed the Kyushu Environmental Evaluation Association in 1971. KEEA was established, with the support of academics, in response to a need for public sector monitoring and analysis of water quality in the aftermath of the emergence of Minamata Disease (KEEA, 2015a, KEEA, 2015b). This initial focus on water quality expanded into other effects of industrialisation on daily life such as air pollution, and KEEA has since diversified further. The organisation commenced planning and design activities in 1989; started climate change countermeasure works in 1999; registered as a construction consultant in 2002; and has hosted the Fukuoka Prefecture Center for Climate Change Actions (a citizens’ climate action network) since 2004 (KEEA, 2011).

KEEA’s embeddedness within the community of institutions producing knowledge on the Kyushu environment is evident in their governance structure. Kyushu University and Fukuoka University scholars (including those who feature prominently in Fukuoka’s climate governance) hold office and sit on KEEA’s advisory board, and some KEEA directors hold positions at Kyushu University (KEEA, 2017). KEEA conversely is represented (through FCCCA) on the Fukuoka City Climate Change Action Plan Committee (Fukuoka City, 2016), and its KEEA board members likewise sit on Fukuoka Prefecture’s Climate Change Action Plan Committee (Fukuoka Prefecture, 2017).

KEEA has since 1971 published the annual *Environmental Evaluation* (*Kankyou Kanri*) journal. This contains scientific articles from KEEA researchers and from other scholars working on environmental issues in Fukuoka and Kyushu more widely, with contributions collated and edited by KEEA. This makes the *Environmental Evaluation* periodicals a useful focal point for tracking the development of the environmental science research and knowledge base among the group of institutions most closely connected to built environment and greenspace governance in Fukuoka City. The *Environmental Evaluation* archives offer an insight into how the empirical evidence base for environmental change management through the built environment has developed over time in the city. The archives also allow us to track how environmental change in Fukuoka has been framed in relation to the broader socio-political context.

## 4 Method

As per Yin (1984), a single case study of this nature has value in contributing to theory-building, more than in generalising across populations. This is in keeping with the study’s overall objectives of furthering scholarly thought on how epistemic communities shape environmental policy in specific domains at the local level; and clarifying the factors which can contribute to effective evidence-based environmental policy formation in particular locations. Content analysis was undertaken on KEEA’s *Environmental Evaluation* journals, covering every year’s report from KEEA’s foundation in 1971 up to the latest edition available (2017) at the time the research was undertaken. This gave a sample of 46 documents, which are publicly available online in Japanese at http://www.keea.or.jp/kankyokanri.html. The value of these journals is that they give insight into how the wider applied environmental science evidence base informing urban environmental policymaking in Fukuoka and Kyushu has evolved over time. Specific to the areas of built environment, greenspace and environmental change which are assessed in this paper, analysis of content in *Environmental Evaluation* allows us to track over time how an epistemic community has emerged in Fukuoka. Specifically, it yields insight into (a) the key techno-scientific knowledge base identified; (b) how the need for and purpose of this knowledge is rationalised and justified in terms of the benefits it provides to wider society; and (c) what policy actions and forums the epistemic community sees as being effective for inducing change.

A directed content analysis approach (Hsieh and Shannon, 2005) was followed, whereby the content of the documents was analysed and interpreted guided by understanding of the wider context surrounding their production. To this end, other relevant documentation relating to environmental change, the built environment, greenspace, and environmental history in Fukuoka was reviewed. Materials consulted included Fukuoka City’s Climate Change Action Plan (Fukuoka City, 2016); the Fukuoka Prefecture Climate Change Action Plan (Fukuoka Prefecture, 2017); Fukuoka City’s New Green Basic Plan (Fukuoka City, 2009); and the Fukuoka City Urban Planning Masterplan (Fukuoka City, 2014). Relevant academic articles and texts detailing local environmental history were also sampled for additional context. This was done by searching both Web of Knowledge and Google Scholar for peer-reviewed outputs; and searching the Japan Society for the Promotion of Science’s Grants-In-Aid for Scientific Research (KAKENHI) database to identify relevant funded research projects. For both peer-reviewed projects and outputs, the search terms used were Fukuoka (*Fukuoka*); climate change (*kikou hendou*); global warming (*chikyuu ondanka*); urban planning (*toshi keikaku*); urban development (*machizukuri*); greenspace (*ryokuchi*); and urban greening (*ryokka*) to reflect the distinctive features of the Fukuoka case raised in Section 3.1. This was supplemented with visiting the library at Fukuoka City Botanical Gardens (a repository for documentation in the city) for older material not available online.

This broader documentation was used to construct a background picture of the evolution of knowledge about local environment, climate, and its relationship to the lived environment in Fukuoka; refining and clarifying key themes to be evaluated in the *Environmental Evaluation* articles. *Environmental Evaluation* archives were then read firstly for the titles of articles to identify potentially relevant material. Relevant articles were thereafter read in full, reading in particular for mention of a more refined list of terms for greater analytical precision: climate change (*kikou hendou*); global warming (*chikyuu ondanka*); climate change prevention (*chikyuu ondanka boushi*); climate change countermeasures (*chikyuu ondanka taisaku*); climate change adaptation (*chikyuu ondanka tekiou*); urban planning (*toshi keikaku*); urban development (*machizukuri*); greenspace (*ryokuchi*); and urban greening (*ryokka*). These themes reflect our study’s interest in how an epistemic community has emerged in Fukuoka around managing environmental change through the built environment and greenspace, and were chosen to add extra nuance to the search terms used for identifying relevant scientific papers and projects. The supporting policy and academic documentation mentioned above was also re-read for mention of these terms.

Given the limited scholarly material available on urban environmental governance in Fukuoka to date, this directed content analysis – which allows the sampled material to be read in-depth and in relation to its broader context – was considered an appropriate analytical tool to offer insight into a complex topic. Similar techniques for analysing empirical material have been used elsewhere to map out the emergence of environment or climate knowledge within a specific social context (e.g. de Freitas and Dias, 2017, Heidenblad, 2018). Counting set words and phrases within the documents may have been of limited value given that only a small number of documents were available each year. Principles of social science methodological rigour (Teel et al., 2018) were adhered to through noting relevant indicative extracts and presenting these with the analysis; utilising publicly-available sources to allow verification of argumentation (as far as possible URLs to all articles relied on for analysis are provided in the reference list); and assessing/evaluating findings in relation to extant social science theory. Accordingly, relevant extracts in the texts were noted and translated into English, and are used to form the basis of the discussion in 5, 6. The lead author takes full responsibility for all translations.

### 4.1 Limitations

Qualitative content analysis of this nature perhaps inevitably involves an element of interpretation on the part of the researcher. In keeping with best practice in the field, we have sought to demonstrate the robustness of the arguments through presentation of evidence (i.e. quotes) that allow the reader to verify our assertions for themselves, and by making connections to the underpinning social science theory to illustrate how our observations fit in with existing thinking in the field (Mays and Pope, 1995, Henwood and Pidgeon, 2012). Further research may wish to consider building a larger dataset of materials, which would allow trends over time to be quantified and mapped out (e.g. Supran and Oreskes, 2017). This could be done not only for Fukuoka, but also other cities with longer histories of evidence-informed environmental policy. Nevertheless, the issue at hand is complex, and the amount of previous scholarly research into environmental governance, expert knowledge and the planned environment in Fukuoka is limited. We hence believe an approach offering flexibility to view texts holistically is important for starting to make sense of how an epistemic community can shape environmental policy in a specific locality.

## 5 Characterising Fukuoka’s epistemic community for urban environmental change

To characterise and assess the epistemic community informing built environment and greenspace policy for environmental change management in Fukuoka, we consider how each of the characteristics of an epistemic community defined by Haas (1992) is reflected in *Environmental Evaluation* and wider texts and actions over time. Table 1 summarises the key institutions which emerge in our characterisation of Fukuoka’s urban environment epistemic community, and their roles within the environmental governance and policy process. Table 2, meanwhile, provides an overview of the main research activities, policy actions and national and international drivers relating to environmental change in cities over time. Table 2 is an indicative summary of how the research landscape, policy actions and external drivers within which the epistemic community operates has developed over time, to illustrate themes which will be evaluated in more depth in Sections 5.1, 5.2, and 5.3. It is not intended to be an exhaustive summary.

### 5.1 Shared causal beliefs and shared notions of validity

We first assess shared causal beliefs and shared notions of validity. We consider these two elements together, as they strongly overlap in this case. In Fukuoka, shared causal beliefs come through a common concern with modification of the lived urban environment (including greenery and greenspaces) as a means of sustaining quality of life under a number of pressures. Shared notions of validity come through the common use of observations, and also models and simulations, to understand effects of environmental changes on the lived environment. Fukuoka Women’s University Principal Makoto Takagi notes the consistency of these causal beliefs and notions of validity over time, in an editorial on environmental observation and equipment for data analysis:

*“For the carbon dioxide and climate change issues of recent years and the issues of acid rain and yellow dust across oceans, the local issues of 30 years ago have increased in scale but that does not mean the basic observation has changed […] The difficulty is perhaps developing the ‘soft’ technology (social organisation, operational organisation) to operate ‘hard’ technology effectively in society”* (Takagi, 2007: 1-2)

Even when allowing for translation, the use of the phrase ‘soft’ both here and in Imura (1993) to describe social systems gives insight into what kinds of evidence are considered valid drivers of environmental policy in Fukuoka. The significance of techno-scientific knowledge, derived from empirical observation, to management of Fukuoka’s lived environment can be viewed from the early 1980s. Kyushu University Department of Agriculture Professor Tsutomu Sakagami mentions observation of a changing climate – *kikou hendou* – in 1981. He links this to changes in atmospheric composition due to human activity, and considers the effects these observed changes may have on Kyushu’s agriculture (Sakagami, 1981). In the same year, a group of academics from Kyushu Institute of Design published *Design of Micro-Weather in Urban Greening* (Nitta et al., 1981). Nitta et al. argue for a technical and coordinated approach to urban greening in Fukuoka and other Japanese cities, to provide temperature moderation and air purification as opposed to purely aesthetic benefit. In both of the texts described above, empirical observations are used to justify areas of concern to be addressed through policy actions.

The idea that empirical observations of the local environment are an appropriate and necessary base for informing built environment and greenspace policy comes across especially strongly in Fukuoka for one issue – the urban thermal environment. Fukuoka’s activities in this area are sufficiently early and rigorous in comparison to the international context (cf Hebbert and McKilliop, 2013) to warrant particular attention. Research into water bodies (Katayama et al., 1990) and wind corridors (Katayama et al., 1991) in Fukuoka draws on remote sensing observation and field observation of wind speeds respectively to illustrate cooling effects. The connection of such evidence to policy recommendations can be seen in *Environmental Evaluation*, where a 1995 article sets out the value of urban greenspace in regulation of temperature and also rainfall:

*“Thus far, regarding the effect of greenspace on flood control due to rainfall infiltration, there has been guidance considering increase of the flood control effect by creating regulating ponds etc when large-scale developments are happening. However, there are not yet examples of planning to consider mitigation of heat islands. In Karlsruhe in Germany, there are policies to plan greenspace configuration to secure wind corridors. In this way, greenspace is evaluated as a means of solving thermal issues, and even in dense cities it is necessary to create greenspace alongside efficient use of space.”* (Miura, 1995: 34)

The links between the shared causal beliefs and notions of validity which Fukuoka’s built environment epistemic community holds on one hand, and the actual policy recommendations and decisions for cooling via greenspace on the other, become even clearer into the 1990s and 2000s. Yoda and Katayama (1998) conducted a study of climate analysis in Fukuoka for urban planning. One of the authors, Tadahisa Katayama, was based at Kyushu University and has undertaken research into the urban thermal and wind environment in the city since at least the 1980s (e.g. Katayama et al., 1990). Thereafter, Fukuoka’s 1999 greenspace plan refers to the protection of wind corridors and to the role of greenery in cooling at the building level, with the plan formation committee chaired by an academic from Kyushu Institute of Design and having representation from Kyushu University (Fukuoka City, 1999). There is hence good crossover between the observation-driven activities of key research institutions, and Fukuoka City’s policy outputs at the time.

Thereafter, the knowledge base within Fukuoka for consideration of climatic issues within urban- and greenspace planning further develops. This reflects increasing political significance of environmental change in Japan through, for instance, the enactment of the Kyoto Protocol in 2005. Fukuoka was one of the first two Japanese cities (alongside Tokyo) to produce an urban climatological atlas focusing on climate functions and greenspace. Land cover and energy consumption surveys for Fukuoka were produced in 1998 as the basis for an urban climate atlas (Yoda and Katayama, 1998). An urban climate atlas for Fukuoka was subsequently published in 2003 under the guidance of the Ministry of Land, Infrastructure and Transport (Ichinose et al., 2003), citing Tadahisa Katayama’s work. Fukuoka City Government’s research and planning around urban heat shows further refinement through building- and neighbourhood-scale research into cooling effects undertaken by researchers from KEEA, Kyushu University and Kyushu Institute of Technology among others (Tanaka, 2009, Tanaka et al., 2009). Findings and outputs from these studies are directly cited in Fukuoka City’s New Green Basic Plan, in which ‘hot’ areas of Tenjin and Hakata Station are identified as priority sites for cooling measures (Fukuoka City, 2009).

Shared causal beliefs and notions of validity are not limited to thermal environments. The aspiration to influence urban policy and practice through the provision of observation-derived scientific ‘evidence’ is stated in recent *Environmental Evaluation* papers about Fukuoka more broadly. In articles drawing on quantitative and/or spatial data, both Ooi (2008) on urban biodiversity conservation, and Takashi (2013) on preservation of urban rivers, justify their work as providing evidence for development decisions. Academics from Kyushu Institute of Technology, Fukuoka University and Kyushu University have also worked with communities and non-government stakeholders to provide technical advice for decision-making on flood responses (Yamashita et al., 2016).

This section illustrates how a community of institutions and scholars in Fukuoka has sought to frame observation-based environmental science as the basis for informing local policy on managing change through the built environment. This is linked to the group’s own interests in observing environmental change in the built environment, and their application of these techniques to Fukuoka itself. Such influence is particularly prevalent with regard to the urban thermal environment, but is also evident in other environmental change issues such as flooding and biodiversity. To understand how these scholars and institutions inform local policy with their knowledge, we now evaluate the processes through which the community seeks influence.

### 5.2 Common policy enterprise

We now assess the common policy enterprise of the community of scholars working on environmental change management through built environments and greenspace in Fukuoka. We assess how the outcomes of the research outlined in Section 5.1 are aligned to the city’s broader environmental governance trajectory. In Fukuoka, the epistemic community seems able to shift the framing of their activity to connect their actions to national and international political trends around environmental issues. Yet this community retains a consistent focus on a liveable environment, and on lived/planned environment policy as a site for attaining this influence.

Across the *Environmental Evaluation* outputs, the epistemic community in question is able to connect built environment and greenspace management to a breadth of issues associated with environmental change. These links increase in specificity and complexity over time. The value of planning is raised, for instance, in the early 1980s in relation to local weather patterns and environmental quality:

*“(F)or example we need to formulate a green masterplan, which is a city oasis aiming to provide water (air moisture) and improve air quality. We also need to evaluate and create a healthier environment for living space, where natural and artificial are merged as one.”* (Sakagami, 1981: 49)

Planning is then raised in the 1990s, in the context of reconciling socio-economic development pressures with climate change and environmental issues:

*“However, in recent years in urban environmental issues, environmental elements which have not previously been taken into account have become issues, and conventional techniques are becoming no longer able to deal with these sufficiently. This primarily started to appear in the heat island issue, which has then become more evident in global environmental issues.”* (Miura, 1995: 32)

In the 2000s, planning emerges again in relation to new and potentially more complex urban environmental issues such as biodiversity conservation:

*“To plan an urban development where in the gardens and street trees birds sing, butterflies dance and we live alongside the natural environment, it is necessary to sustain forests and cultivated land within the city. And by using a habitat model, we can decide the best arrangement...”* (Ooi, 2008: 35).

These extracts illustrate that influencing planning and management of the built environment in response to environmental change has remained a constant policy enterprise over time for this particular environmental science-based epistemic community. Yet this community is able to shift and adapt their policy enterprise to overarching rationales over time. From the mid-1980s, Fukuoka University Professor Naohito Asano argued that as pollution issues in the local environment became resolved, the preoccupation of local scholars shifted towards urban environmental planning and the provision of a ‘liveable environment’ (Asano, 1988). This idea of *kaiteki kankyou* – a liveable environment – can be seen across Fukuoka at this time. *Kaiteki kankyou* appears in a 1985 contribution by Kenji Mitsuyoshi of Kyushu University titled *The city and a liveable environment*, and the phrase is used in Fukuoka City’s environmental plan (Fukuoka City, 1986). The goal of a liveable environment was similarly included in the 1985–87 project ‘Land Use and Sustaining and Improving the Environment in Cities and their Surroundings’, in which Asano was a participant and through which land use change in Fukuoka was mapped through aerial photography to understand problematic issues and recommend new strategies for urban land use policy.

This framing shifts into the 1990s towards a greater emphasis on urban sustainable development, and to the local environment as the site at which global environmental issues manifest themselves yet can also be managed. Research from Fukuoka at this time carries the sense that ‘conventional’ approaches to planning may be inadequate to address this complexity, and that new forms of knowledge are required to balance environment and development. For instance, Imura (1993) makes reference to the UN Framework Convention on Climate Change, the Rio Declaration, and Agenda 21 in an article on balancing environment and development at the local level. Kyushu University led a research project (1996–1998) into the arrangement of urban green spaces based on function of green spaces, which involved developing a basic green plan in Munakata City within Fukuoka Prefecture (Setsu, 1998). Kyushu Institute of Design led another project (1996–1998) into thermal effects of green areas on the urban living environment (Ishii et al., 1998). Fukuoka City’s Green Basic Plan of 1999 reflects this emerging framing of urban environmental problems as complex issues where new knowledges and skills are required to balance different and sometimes competing pressures. The plan lists climate change, acid rain, the ozone layer, and species extinction as factors ultimately affecting daily life which must be considered in greenspace provision within the city. The plan also considers the relationship of greenspace to water provision, recycling and energy consumption (Fukuoka City, 1999). Across these examples, the epistemic community seeks to position planning, greenspace and underpinning techno-scientific evidence as necessary to respond to these challenges.

The most recent framings of built environment and greenspace scholarship in Fukuoka situate the quality of the local environment as integral to development, rather than as a barrier to progress. The idea of thinking in terms of ‘green resources’ rather than ‘green space’ was suggested by Asano (1988). *Environmental Evaluation* contributions have since applied this more holistic way of thinking to a breadth of environmental issues. These include Ooi (2008) on understanding the value of ecosystems as a basis for conservation; Asano (2017) on environmental evaluation in the context of attaining the UN Sustainable Development Goals; and Hijioka (2017) on integration of climate change adaptation actions with existing environmental and disaster prevention policies. These research outputs reflect environmental policy trends in Fukuoka, notably the integration of climate adaptation and mitigation with wider environmental planning in Fukuoka City’s 2016 climate change plan; and the initial consideration of ecosystem services in Fukuoka Prefecture’s 2017 climate plan.

There appear to be two key ways in which Fukuoka’s epistemic community exerts influence over governance of planning and greenspace to steer these processes in the direction of managing environmental change. One is through participation in local expert committees in relevant policy fields, for instance climate change, greenspace, and urban planning. Both the city- and prefectural-level climate change plans have input from Prof Naohito Asano, whose lengthy historical involvement with issues of the urban environment and environmental law in Fukuoka are documented above. The Fukuoka City Urban Planning Masterplan; Fukuoka City Climate Change Countermeasures Action Plan; and Fukuoka Prefecture Climate Change Countermeasures Action Plan committees all include scholars today who work in the same departments as key figures from the city’s urban climate and greening studies did in the 1980s and 90s. Such committees provide a forum through which expertise and research findings may shape policy, and in the case of Fukuoka have over time received input from the same network of scholars. The second way in which the epistemic community exerts influence is through collaborative projects with the city and national government. As outlined in Section 5.1, research into issues such as heat mitigation (Tanaka et al., 2009) and more recently flood risk management (Yamashita et al., 2016) has been undertaken in collaboration with the city government. These projects have had the explicit intention of providing an evidence base to inform city government decisions about managing environmental risk and change through the built environment.

### 5.3 Shared normative belief

Linked to a common policy enterprise is the question of why the epistemic community forming around environmental change and the built environment in Fukuoka seeks to engage with policy. The institutions and actors involved appear to have a shared normative belief in the preservation of local living quality for citizens. This is facilitated by rigorous environmental science undertaken in the public interest to evidence judgements of environmental quality and justify recommendations for countermeasures through policy.

To understand why this shared normative belief remains relevant to the epistemic community today, it is necessary to look to the local historical context. Specifically, the activities of the epistemic community’s member institutions and individuals in producing techno-scientific knowledge in response to earlier environmental issues and their governance in Fukuoka. The shared normative belief at least partly has its roots in negative experiences with pollution experienced in the vicinity of Fukuoka. The 1950s saw the initial identification of Minamata Disease, named after the town in Kyushu south of Fukuoka where discharges of poisonous metals by the Chisso Corporation into the sea caused severe illness and birth defects among citizens over several decades. The Minamata incident gave rise to concerns over the negative side-effects associated with Japan’s rapid economic growth, and to social and environmental justice claims (Iijima, 1994). Not long after, the city of Kitakyushu north of Fukuoka became a site for contestations over air and water pollution from industrial activity, with citizen action pressing the government and industry to adopt stricter standards (Fujikura, 2001).

The historical motivation of scholars in Fukuoka to first engage with environmental issues is narrated in KEEA’s organisational overview:

*“‘Minamata Disease’ which was discovered in 1956 is today known widely across the world. At that time, to prevent pollution of public waters, monitoring by a public organisation was desired […] Based on this need, in 1970 with the commitment of two university professors the Kyushu Water Quality Analysis Research Institute was established […] To extend the public mission of our research organisation beyond water contamination to look at air contamination and many other kinds of pollution, we started our full-scale operations as the Kyushu Environmental Evaluation Association independent foundation.”* (KEEA, 2015b)

The role of university professors in founding KEEA as a provider of independent and public-facing scientific research indicates the commitment of individuals working within institutions in Fukuoka to the protection of local environmental quality. However, once water and air pollution issues in northern Kyushu reduced in severity, many of these institutions and indeed individuals turned their attention to the planned environment and environmental change. Despite being formed to address water quality issues, KEEA today hosts the Fukuoka Center for Climate Change Actions, undertakes urban thermal environment research in collaboration with the city government (Tanaka et al., 2009), and holds a construction consultant’s licence as well as urban planning expertise. The transition of a community of researchers and institutions from an interest in pollution to an interest in securing a liveable environment for citizens – the *kaiteki kankyou* discussed in Section 5.2 – is evident in *Environmental Evaluation* too. This can be seen in articles in the mid-1980s from Kyushu University Department of Engineering Professor Kenji Mitsuyoshi, and Fukuoka University Department of Law Professor Naohito Asano:

*“(T)he shadow of industrial pollution has almost been hidden, but the pollution from people’s daily lives is remarkable […] Preparing for urban living is necessary, but this does not just mean securing housing. The provision of a comfortable environment through maintenance of open spaces such as parks, green space etc is required”* (Mitsuyoshi, 1985: 4-8)

*“Arguing for a ‘liveable environment’ offers a new way to think about environmental issues that goes beyond this framing of ‘pollution and nature.’”* (Asano, 1988: 14)

The role of Asano here is significant given his involvement with both victims of Minamata Disease (Asano, 1992b) and subsequently environmental and climate change planning in Fukuoka from the 1980s to present. The histories of figures such as Asano and institutions like KEEA illustrate how the competences and normative beliefs of key people and organisations in Fukuoka have carried over from pollution to built environment issues, where they have shaped an epistemic community focused on responding to environmental change. Even more technical actions reported in *Environmental Evaluation* and elsewhere, such as preservation of wind corridors (Nitta et al., 1981) and city-scale climatological planning (Miura, 1995), are justified in terms of bringing quality of life to citizens. As such, built environment and greenspace issues tap into a bigger narrative and normative belief in Fukuoka of environmental science for the benefit of citizens, forming a basis and justification for the epistemic community to seek to influence policy.

At a personal level, there is also significant investment of Fukuoka-based scholars in the quality of the environment in which they live. This is illustrated by the engagement of researchers with various expert committees and government projects within the city, as outlined in Sections 5.1 and 5.2. A number of the scholars involved in built environment and environmental change research in Fukuoka have been born and/or educated in the city; and particular departments within institutions have remained engaged with policy over time as research activity has passed across generations of scholars.^1^ This gives sustained engagement of a core community of scholars with the local government in Fukuoka over time, grounded in a consistent methodological and normative base. Present-day environmental change actions, especially the climate change countermeasures described in Section 3, may hence be viewed as just the latest in a number of iterations of citizen wellbeing through urban environmental policy and underpinning research. Indeed, whilst writing about Japan more generally, National Institute of Environmental Studies scholar Yasuaki Hijioka reflects that:

*“Our country’s adaptation actions are still just at the startline, but by bringing together a long history of experience, technology and knowledge in protecting citizens’ livelihoods in areas such as disaster prevention, farming, health etc, I hope that industry, academia, the government, and the public can work together to progress towards a safe and secure future society.”* (Hijioka, 2017: 24)

There is, therefore, a constant normative core of science in the public interest to protect citizen welfare. Present-day expert-driven actions to manage environmental change via planning and greenspace in Fukuoka therefore fit with the knowledge systems established to address air and water pollution issues in the 1970s and 1980s, and also link to an overarching research objective of sustaining living quality for the residents of Fukuoka and Kyushu. This normative agenda may go some way to explaining both the research competences and motivations of local institutions and the people within them to understand different iterations of change in the built environment, and to seek to inform urban environmental policy.

## 6 Discussion and conclusions: significance and limitations of epistemic communities at the local level

### 6.1 What contributes to evidence-driven policy in Fukuoka, and what is distinct about local-level epistemic communities?

In response to challenges raised in extant literature (e.g. Lane et al., 2008, Hughes and Romero-Lankao, 2014, Acuto, 2018), we reflect on what is different about how epistemic communities operate for environmental issues at the city level, as opposed to the international level at which the concept originally emerged. We propose four factors which contribute to effective alignment between an epistemic community and environmental policy at the city level. First is *historical context*. As discussed in Section 5.3, Fukuoka has experience with water and air pollution in the surrounding area. The subsequent need for independent scientific data to evidence claims against polluting companies strengthened a community of scholars with competences in environmental assessment, committed to informing local environmental policy with evidence to ensure citizen wellbeing. This gives a strong narrative and historical base, into which urban planning and greenspace management to counter environmental change can fit. In other cases where urban environmental policy in the present is informed by techno-scientific evidence, the underpinning competences can likewise be traced back to previous environmental issues. This resonates with findings from Durban, where ecological knowledge emerging in the 1970s for conservation purposes was later applied to ecosystem services and an urban open space system (Freund, 2001). The Fukuoka case also parallels the origin of urban climatological planning in German cities such as Stuttgart, which has its roots in understanding of air flows gained through military activity and subsequently applied to pollution matters prior to consideration of climate issues (Hebbert and McKilliop, 2013).

Second is the *strong personal investment of the epistemic community in the city itself.* Many individuals within the epistemic community work and live within Fukuoka City, with some of them (particularly the current generation of practitioners) born in Fukuoka and graduating from the city’s institutions. The producers of key texts in the 1980s and 90s have taught or supervised the current generation of urban environment and climate scholars who inform the city and prefecture’s planning committees. Third and related is the *accessibility of the epistemic community to wider society* via engagement. KEEA had its origins in the commitment of Kyushu University academics to provide independent data for local society, and today is responsible for public-facing engagement on climate issues in the city through its hosting of the Fukuoka Center for Climate Change Actions. Meanwhile, Naohito Asano, an active figure on municipal and prefectural climate change planning committees, had prior involvement with victims of Minamata Disease. This sets a context for scholars working with and for wider society and not in isolation. Moreover, given the relatively low inequality and high access to education (Fukuoka City Statistics, 2019) within Fukuoka, it is also the case that the local institutions at the core of the epistemic community – Kyushu University, Kyushu Institute of Design, Fukuoka University – may be considered relatively accessible to much of the population. This reflects not only Haas’ idea of the epistemic community having a normative core, but also Ozdemir’s (2018) argument that ‘experts’ are not necessarily apolitical and can steer environmental policy in response to their interactions with citizens and communities.

Fourth and final, it is important to acknowledge that even within Japan, Fukuoka is a *wealthy and well-resourced city*. This allows the city on occasion to invest in research, and also gives it access to the breadth of competent institutions and expertise required to enact expert-driven policy formation. Whether a favourable historical context and the strong commitment of an epistemic community would be enough to enable an expert community to shape local environmental policy in other, less well-resourced, subtropical Asian cities is a question that ought to be explored through further research.

Moreover, the epistemic community in Fukuoka has not completely succeeded in shaping local planning and greenspace policy to mitigate environmental change. There are of course successes, where studies in urban thermal environments and extreme rainfall have informed small-scale cooling countermeasures and rainfall reservoirs. This is evident in, for example, connection to thermal environment research in the Fukuoka City New Green Basic Plan (2009), and mention of green infrastructure research in the Fukuoka Prefecture Climate Change Countermeasures Action Plan (2017). However, challenges remain, such as the blocking of wind corridors through previous development (Katayama et al., 1991) and difficulties in synthesising advice across government sectors (Mabon et al., submitted for publication).

### 6.2 Value and limits of epistemic communities to urban environmental policy and governance

This final subsection reflects on what Fukuoka tells us about epistemic communities and their analytical value in understanding contemporary urban environmental issues. We return to Toke’s (1992) assertion that it may be impossible to avoid making normative judgements for environmental issues, and consider the implications of this for seeing epistemic communities as a higher authority for setting appropriate environmental policy. There is of course a strong public interest narrative running through how actors like KEEA justify their scientific activity. Yet when it comes to societal context, the input of the epistemic community in Fukuoka has tended more towards the ‘technocratic’ governance that Stephens and Liu (2012) warn of. The role of the public and civil society in Fukuoka in this context appears to be to receive ‘information’, and respond to directives issued by the local government. KEEA’s periodicals refer to social factors as ‘soft’ systems to be utilised to put techno-scientific ideas into practice (Imura, 1993, Takagi, 2007), and civil society engagement is mentioned in *Environmental Evaluation* mainly as reportage on public outreach activities of FCCCA.

Moreover, focusing on the scientific community and its influence on climate and environment policy may overlook the role that civil society actors have played in Kyushu in managing the urban environment and driving the environmental governance agenda. The push for establishment of organisations such as KEEA, who today form part of the epistemic community around the planned environment in Fukuoka, came via calls from citizens for evidence-based regulations against industrial pollution. In cases citizens have produced the requisite data themselves, as per Takeoka Chatfield and Reddick (2016) on first pollution control and then smart city implementation in Kitakyushu. It is especially worth noting the role women played in pushing for a rigorous independent evidence base to respond to the pollution incidents of the 1960s (Fujikura, 2001). Furthermore, practices such as *uchimizu* – the sprinkling of water on streets and gardens for summer cooling, dust reduction and aesthetic benefit – have recently been promoted by academic institutions and strategised in Fukuoka’s climate action plan for their heat risk reduction potential (Fukuoka City, 2016, Fukuoka Institute of Technology, 2018). Yet these practices have their origins in citizens’ daily practice (not only in Fukuoka but also across all Japan), pre-dating the epistemic community characterised in this paper. These narratives may lie outside formalised policy structures, yet are crucial to the success of the epistemic community by setting a context in which management of the lived environment and environmental regulation is seen as desirable for local society.

The idea of epistemic communities, especially when understood at the local scale, may be a useful conceptual tool to understand how evidence-based urban environmental policy for specific issues can come to evolve in specific locales. Thinking in terms of epistemic communities can illustrate ways in which scientists may act to ensure local environment and climate policy is informed by the robust evidence base required for what are very real and harmful risks. Yet whilst there is no disputing the importance of formal research and policy processes, Fukuoka’s environmental history shows that civil society groups are important in raising the profile of local environmental issues as areas of concern. In other words, the normative convictions of members of the epistemic community alone might not be enough to explain how and why localised urban environmental policies have taken root. Following Toke, 1992, Pieterse, 2006, then, we suggest a strong and reflexive epistemic community is a valuable part of understanding an appropriate response to current urban environmental challenges. However, this ought to be tempered with input from environmental and/or civil society groups who may be better placed to bridge this evidence base with the social justice issues which are increasingly seen as key to environmental governance in cities, yet lie beyond the scope or purpose of a science-driven epistemic community. Such ideas of co-creation, co-production and the integration of scientific with traditional and local knowledges are indeed acknowledged in the body of work at the urban science-policy interface (e.g. Prieur-Richard et al., 2018). Our findings resonate with this turn, and serve as a reminder that a strong community of experts from academia and research is but one part of an effective evidence-driven response to environmental challenges in cities.

## Footnotes

1 We do not feel it is appropriate to single out specific individuals or departments by naming them here. However the lists of expert committee members can be found in the relevant policy documents, and our observations are based on basic biographical information and academic histories publicly-available on academics’ own websites.
